# [^111^In]PSMA-I&T: expanding the spectrum of PSMA-I&T applications towards SPECT and radioguided surgery

**DOI:** 10.1186/s13550-015-0147-6

**Published:** 2015-11-25

**Authors:** Margret Schottelius, Martina Wirtz, Matthias Eiber, Tobias Maurer, Hans-Jürgen Wester

**Affiliations:** Chair for Pharmaceutical Radiochemistry, Technische Universität München, Walther-Meissner-Strasse 3, 85748 Garching, Germany; Department of Nuclear Medicine, Klinikum rechts der Isar, Technische Universität München, Ismaningerstr. 22, 81675 Munich, Germany; Department of Urology, Klinikum rechts der Isar, Technische Universität München, Ismaningerstr. 22, 81675 Munich, Germany

**Keywords:** Prostate-specific membrane antigen, PSMA, PSMA-I&T, Imaging, PET, SPECT, ^111^In, ^177^Lu, Targeted radionuclide therapy, Radioguided surgery

## Abstract

**Background:**

The relevance of prostate-specific membrane antigen (PSMA) targeting in the clinical management of prostate cancer (PCa) is continually increasing, entailing the development of PSMA-targeted molecular probes. Recently, a first PSMA-targeted theranostic concept has been successfully implemented by [^68^Ga/^177^Lu]PSMA-I&T. To further exploit the excellent PSMA-targeting characteristics and in vivo performance of the PSMA-I&T platform, [^111^In]PSMA-I&T was evaluated as a complementary probe for radioguided surgery and SPECT imaging.

**Findings:**

Compared to [^68^Ga/^177^Lu]PSMA-I&T, [^111^In]PSMA-I&T showed unchangedly high PSMA-affinity and enhanced internalization into PSMA-expressing LNCaP PCa cells. Biodistribution studies in LNCaP xenograft-bearing mice (1 h p.i.) revealed slightly reduced background accumulation of [^111^In]PSMA-I&T compared to [^177^Lu]PSMA-I&T and identical tumor uptake of both compounds, leading to increased tumor/background ratios for [^111^In]PSMA-I&T. An exemplary patient with metastatic PCa underwent preoperative [^68^Ga]HBED-CC-PSMA PET/CT (1 h p.i.) and [^111^In]PSMA-I&T SPECT/CT (4 h p.i.), followed by prostatectomy and radioguided extended pelvic lymphadenectomy (24 h p.i.). In [^111^In]PSMA-I&T SPECT/CT, the previously identified PCa lesions ([^68^Ga]HBED-CC-PSMA PET/CT) showed high tracer accumulation and were also detectable using planar scintigraphy. The intraoperative use of a hand-held gamma probe allowed detection and resection of all [^111^In]PSMA-I&T-accumulating lesions. The presence of PSMA-positive tumor tissue in the resected specimens was confirmed histopathologically and via [^111^In]PSMA-I&T autoradiography.

**Conclusions:**

[^111^In]PSMA-I&T shows efficient PSMA targeting in vitro and in vivo, combined with low background accumulation. In an exemplary PCa patient, [^111^In]PSMA-I&T was successfully applied for preoperative SPECT/CT visualization and radioguided resection of PSMA-positive lesions, hinting towards a high value of [^111^In]PSMA-I&T as a complementary tool to [^68^Ga/^177^Lu]PSMA-I&T in the clinical management of prostate cancer.

## Findings

Modern clinical management of prostate cancer increasingly relies on exploiting the prostate-specific membrane antigen (PSMA) as a molecular target both for imaging and for treatment of prostate cancer (PCa). PSMA is abundantly expressed on the surface of prostate cancer cells and within the neovasculature of other solid tumors, with limited expression in most normal tissues, establishing the basis for selective targeting of PCa lesions with PSMA-targeted agents. Among the rapidly increasing number of high-affinity PSMA ligands, ranging from intact antibodies to low-molecular-weight compounds, urea-based inhibitors have been most extensively leveraged, with expanding clinical use [1].

Recently, a successful theranostic concept has been realized by the development of [^68^Ga/^177^Lu]PSMA-I&T [2]. Compared to first DOTA-conjugated EuK(=Glu-urea-Lys)-based inhibitors [3], the DOTAGA-conjugate PSMA-I&T has been optimized with respect to PSMA affinity and in vivo stability [2, 4]. First patient studies have demonstrated excellent PSMA targeting for [^68^Ga/^177^Lu]PSMA-I&T, permitting high-contrast PET imaging of metastatic PCa with [^68^Ga]PSMA-I&T and, based on suitable uptake and retention characteristics, efficient treatment with its therapeutic analog [^177^Lu]PSMA-I&T [5, 6].

**Fig. 1:**
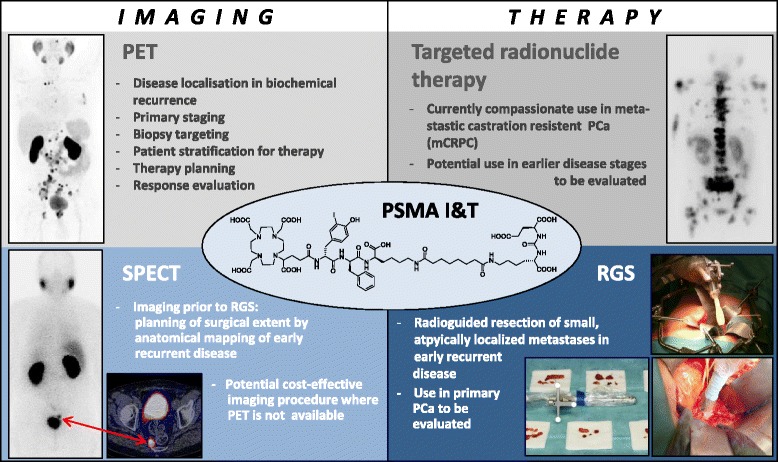
Schematic representation of the PSMA-I&T-based theranostic concept for the clinical management of PCa

The success and ease of implementation of this theranostic approach, relies, among other factors, on the ability of DOTAGA to form stable complexes with a broad variety of radiometals [7] and on the negligible influence of radiometal exchange on the PSMA affinity of [*M^3+^]PSMA-I&T (Table 1) [2]. In the present investigation, these characteristics were exploited to meet the clinical need for a corresponding gamma-emitting probe, which on the one hand allows for the intraoperative detection and identification of PSMA-positive tissues during surgery in patients with early recurrent or primary advanced PCa and on the other hand may additionally be employed for (preoperative) SPECT imaging (Fig. 1). Given the suitable radionuclide characteristics of ^111^In (t_½_ = 2.8 d, E(γ) = 173, 245 keV) for the intended applications, PSMA-I&T was labeled with ^111^In using a standard protocol and was evaluated preclinically and in a first patient.

**Table 1:** PSMA affinities, internalization, and lipophilicity of In-, Ga-, and Lu-PSMA-I&T

Ligand	IC_50_ [nM]	Internalization [% of reference]	Lipophilicity [log P_OW_]
In-PSMA-I&T	7.5 ± 1.5	104 ± 7	−4.5
Ga-PSMA-I&T	9.4 ± 2.9	59 ± 2	−4.3
Lu-PSMA-I&T	7.9 ± 2.4	76 ± 2	−4.1

PSMA affinities were determined in a competitive binding assay using LNCaP prostate cancer cells and (^125^I-BA)KuE as radioligand [2]. Data represent means ± SD of *n* ≥ 3 separate determinations

PSMA-specific ligand internalization was determined by incubation of LNCaP cells (37 °C, 60 min) with the respective radioligands (0.2 nM) in the absence (total internalization) and presence (non-specific internalization) of 10 μM PMPA. Data were corrected for non-specific internalization and normalized to the specific internalization observed for the reference compound (^125^I-BA)KuE in a parallel experiment [2]. Data are means ± SD (*n* = 3)

Lipophilicities (from n-octanol/PBS partition coefficients P_OW_) were determined using a shake-flask method; values are means from *n* = 6 determinations

As anticipated, [^nat^In]PSMA-I&T shows unchangedly high PSMA affinity in a competitive binding assay (IC_50_), which equals that of its ^nat^Lu-counterpart. Unexpectedly, however, internalization efficiency of [^111^In]PSMA-I&T into LNCaP prostate cancer cells was found to be markedly enhanced compared to [^177^Lu]PSMA-I&T (Table 1).

**Fig. 2:**
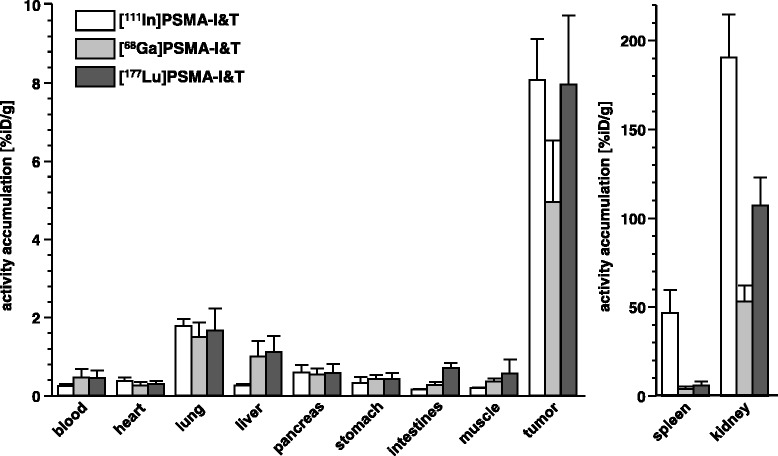
Comparative biodistribution of [^111^In]- and [^177^Lu]PSMA-I&T in LNCaP tumor-bearing mice 1 h p.i. Biodistribution studies were carried out using LNCaP xenograft-bearing CB17 SCID ([^111^In]PSMA-I&T) or CD-1 nu/nu ([^177^Lu]PSMA-I&T) mice. Animals were injected intravenously with 1.4 MBq (0.2 nmol) of the respective PSMA-I&T analog. Data are represented as % injected dose per gram tissue (%iD/g) and are means ± SD (groups of *n* = 5). Animal experiments were conducted in accordance with the German Animal Welfare Act (Deutsches Tierschutzgesetz, approval no. 55.2-1-54-2532-71-13)

In the case of [^68^Ga]- and [^177^Lu]PSMA-I&T, the increased internalization of [^177^Lu]PSMA-I&T, which correlates with its improved PSMA affinity compared to [^68^Ga]PSMA-I&T, was reflected in increased tracer uptake in PSMA-positive tissues in vivo [2]. For [^111^In]PSMA-I&T, however, the observed increase in PSMA-specific cellular uptake compared to [^177^Lu]PSMA-I&T in vitro has no detectable influence on PSMA targeting in vivo. Instead, [^111^In]PSMA-I&T and [^177^Lu]PSMA-I&T show nearly identical uptake in PSMA-positive LNCaP tumor xenografts at 1 h p.i. (Fig. 2), in accordance with the identical PSMA affinity of both compounds.

Besides high tumor uptake, [^111^In]PSMA-I&T also shows significant accumulation in tissues with endogenous PSMA expression, i.e., lung and in particular kidney and spleen (Fig. 1). For the latter two organs, the substantially increased [^111^In]PSMA-I&T uptake is not the result of altered targeting characteristics of the tracer compared to [^177^Lu]PSMA-I&T, but is rather caused by the use of alternative mouse strains for the comparative evaluation of both compounds (CB17 SCID mice for [^111^In]PSMA-I&T vs CD1 nu/nu mice for [^177^Lu]PSMA-I&T). Particularly high tracer uptake in spleen and kidney in CB17 SCID mice has been consistently observed in the evaluation of a variety of PSMA-targeted radiopharmaceuticals in our lab and is mouse-strain specific.

In contrast, the accelerated blood clearance and reduced background accumulation of [^111^In]PSMA-I&T, especially in liver and intestines, are tracer specific. Due to its reduced lipophilicity compared to [^68^Ga]PSMA-I&T and [^177^Lu]PSMA-I&T (see Table 1), hepatobiliary excretion is further reduced in favor of almost exclusive renal excretion. Consequently, [^111^In]PSMA-I&T shows improved tumor(t)-to-background ratios compared to [^177^Lu]PSMA-I&T at 1 h p.i., i.e., t/blood-, t/liver-, t/intestines-, and t/muscle-ratios of 34 ± 8, 32 ± 6, 53 ± 8, and 43 ± 6, respectively, versus 18 ± 9, 7 ± 3, 12 ± 3, and 14 ± 9 for [^177^Lu]PSMA-I&T.

**Fig. 3:**
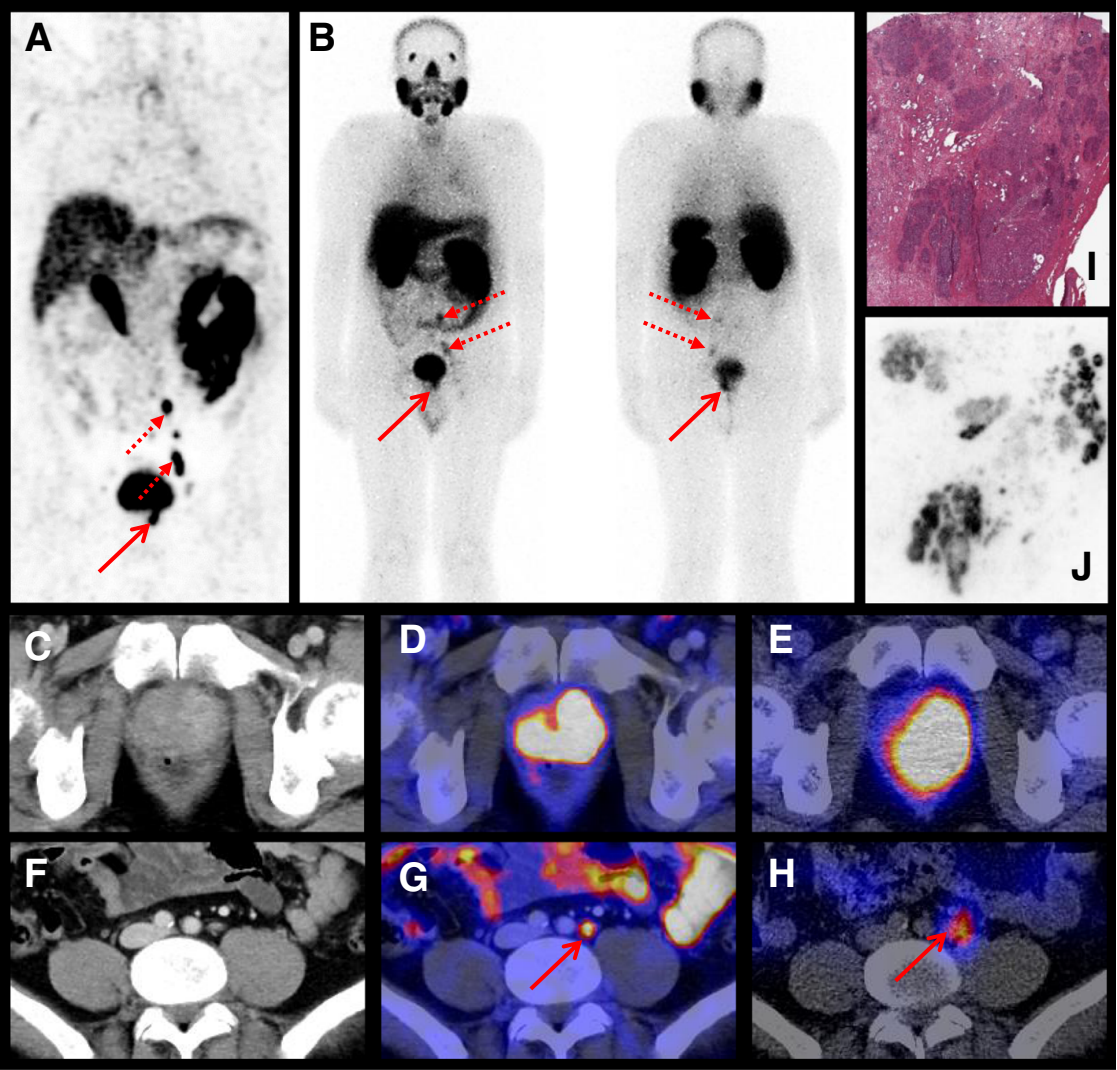
Preoperative imaging using [^68^Ga]HBED-CC PSMA PET/CT and [^111^In]PSMA-I&T SPECT/CT and planar scintigraphy. The human study was approved by the institutional review boards of the participating medical institutions, and the patient provided signed informed consent. **a** [^68^Ga]HBED-CC PSMA PET/CT (MIP) 1 h p.i. **b** planar scintigraphy (ventral and dorsal view) 4 h p.i. of 155 MBq [^111^In]PSMA-I&T. **d**, **g** Axial [^68^Ga]HBED-CC PSMA PET/CT images of the primary tumor in the prostate (**d**) and a representative lymph node (**g**). **c, f**  Corresponding CT images, and **e**, **h** Corresponding axial [^111^In]PSMA-I&T SPECT/CT images of the primary tumor in the prostate (**e**) and a representative lymph node (**h**). **i**, **j** H&E staining and ^111^In-autoradiography of cryosections from resected prostate tissue

Based on these findings, suggesting nearly identical if not slightly improved in vivo PSMA targeting and excretion characteristics for [^111^In]PSMA-I&T in comparison to [^68^Ga]PSMA-I&T and [^177^Lu]PSMA-I&T, an exemplary patient study was carried out to establish the potential of [^111^In]PSMA-I&T as an intraoperative probe for radioguided surgery and to investigate its suitability as a SPECT-imaging agent. The patient (51 years old), presenting with histologically confirmed metastasized prostate cancer (Gleason score 9, initial PSA 63 ng/ml), initially underwent [^68^Ga]HBED-CC PSMA PET/CT for pre-therapeutic staging as previously described [8, 9]. Maximum intensity projection (MIP) showed intense tracer uptake in the primary tumor (Fig. 3a, solid arrow) as well as in multiple pelvic and retroperitoneal lymph node metastases (Fig. 3a, dotted arrows). The patient was scheduled for radical prostatectomy facilitated by radioguided surgery to potentially enhance complete resection of lymph node metastases.

One day prior to surgery, the patient was injected with 155 MBq [^111^In]PSMA-I&T, and preoperative planar scintigraphy as well as SPECT/CT were performed at 4 h p.i. (Fig. 3b, e, h). Radical prostatectomy as well as radioguided lymphadenectomy using a hand-held gamma probe with visual and acoustic feedback (Crystal Probe CXS-SG603; Crystal Photonics, Berlin, Germany) were performed 24 h after injection of [^111^In]PSMA-I&T. The presence of PSMA-positive tumor tissue in the resected specimens was confirmed histopathologically and via [^111^In]PSMA-I&T autoradiography (Fig. 3i, j).

Ventral and dorsal views of whole body planar scintigraphy (Fig. 3b) show intense [^111^In]PSMA-I&T uptake in the primary tumor (solid arrows) as well as in pelvic and retroperitoneal lymph node metastases (dotted arrows). Axial [^111^In]PSMA-I&T SPECT/CT images confirm the intense tracer accumulation, both in the primary tumor (Fig. 3e) and in a representative morphologically not enlarged lymph node (Fig. 3h) and are consistent with [^68^Ga]HBED-CC PSMA PET/CT findings (Fig. 3d, g). During subsequent prostatectomy and salvage lymphadenectomy, the intraoperative use of a gamma probe allowed the detection and quantitative resection of all lymph nodes with [^111^In]PSMA-I&T accumulation. All resected lesions showing tracer uptake were histopathologically confirmed to be metastatic PCa deposits. Within the prostate, ex vivo autoradiography 4 h after surgery showed moderate to intense [^111^In]PSMA-I&T uptake in several intraprostatic tumor foci (I) correlating well with a H&E stained slide from histopathology (J), demonstrating the sensitive detection of PSMA-expressing tumor cells by [^111^In]PSMA-I&T.

Based on these initial promising results, the concept of radioguided lymphadenectomy in early recurrent prostate cancer patients using [^111^In]PSMA-I&T as a PSMA-targeted intraoperative probe has recently been further pursued [10, 11]. In a small cohort of patients, [^111^In]PSMA-I&T radioguided surgery was shown to represent a valuable technique for the intraoperative detection of small subcentimeter metastatic lymph nodes and atypically located lesions.

Additionally, [^111^In]PSMA-I&T showed promising in vivo characteristics as a PSMA-targeted SPECT imaging probe, including suitable whole-body clearance, predominant renal excretion, and efficient accumulation in PSMA-expressing tissues. Of course, in a direct comparison, [^68^Ga]HBED-CC PSMA PET/CT appears as the clearly superior imaging modality compared to [^111^In]PSMA-I&T SPECT/CT. However, this comparison is strongly biased in favor of [^68^Ga]HBED-CC PSMA PET/CT due to the inherent instrumental differences between PET and SPECT with respect to sensitivity and resolution. Furthermore, the comparably high gamma energy of ^111^In further challenges spatial resolution of [^111^In]PSMA-I&T SPECT/CT.

However, the present preclinical and first clinical data underline the suitability of PSMA-I&T as a versatile labeling platform for a variety of PSMA-targeted applications in nuclear oncology and urology, ranging from PET and SPECT to endoradiotherapy and radioguided surgery, encouraging the ongoing further investigation of [^68^Ga]PSMA-I&T, [^177^Lu]PSMA-I&T, and [^111^In]PSMA-I&T as valuable tools for their respective dedicated application.
